# Synaesthesia and sexuality: the influence of synaesthetic perceptions on sexual experience

**DOI:** 10.3389/fpsyg.2013.00751

**Published:** 2013-10-16

**Authors:** Janina Nielsen, Tillmann H. C. Kruger, Uwe Hartmann, Torsten Passie, Thorsten Fehr, Markus Zedler

**Affiliations:** ^1^Department of Clinical Psychiatry, Social Psychiatry, and Psychotherapy, Hannover Medical SchoolHannover, Germany; ^2^Department of Psychiatry, Harvard Medical School, Harvard University, BostonMA, USA; ^3^Department of Neuropsychology, Center for Advanced Imaging Bremen/Magdeburg, Center for Cognitive Sciences, University BremenBremen, Germany

**Keywords:** synaesthesia, sexuality, sexual satisfaction, sexual appetence, oceanic boundlessness, visionary restructuralization, sexual trance, OAVAV

## Abstract

**Introduction**: Synaesthesia is a phenomenon in which a certain stimulus induces a concurrent sensory perception; it has an estimated prevalence of 4%. Sexual arousal as an inducer for synaesthetic perceptions is rarely mentioned in the literature but can be found sometimes in case reports about subjective orgasmic experiences.

**Aims:** To examine whether synaesthetic perceptions during sexual intercourse have an impact on the sexual experience and the extent of sexual trance compared to non-synaesthetes.

**Methods:** In total, 19 synaesthetes with sexual forms of synaesthesia (17 female; 2 male) were included as well as corresponding control data of 36 non-synaesthetic subjects (*n* = 55). Two questionnaires were used to assess relevant aspects of sexual function and dysfunction (a German adaption of the Brief Index of Sexual Functioning, KFSP) as well as the occurrence and extent of sexual trance (German version of the Altered States of Consciousness Questionnaire, OAVAV). Additionally qualitative interviews were conducted in some subjects to further explore the nature of sexual experiences in synaesthetes.

**Main Outcome Measures:** Sexual experience and extent of sexual trance during intercourse.

**Results:** Synaesthetes depicted significantly better overall sexual function on the KFSP with increased scores for the subscale “sexual appetence” but coevally significant lower subscale scores for “sexual satisfaction.” Sexual dysfunction was not detected in this sample. Synaesthetes depicted significantly higher levels of the subscales “oceanic boundlessness” and “visionary restructuralization” than controls using the OAVAV. Qualitative interviews revealed varying synaesthetic perceptions during the different states of arousal. Furthermore, synaesthetes reported an unsatisfactory feeling of isolation caused by the idiosyncratic perceptions.

**Conclusions:** Synaesthetes with sexual forms of synaesthesia seem to experience a deeper state of sexual trance without, however, enhanced satisfaction during sexual intercourse.

## Introduction

Synaesthesia is a neural condition in which a certain stimulus, called inducer, elicits a concurrent perception; for example, listening to music can induce the visual perception of colored patterns or letters and numbers are perceived in specific idiosyncratically associated colors. Current estimates consider synaesthesia to be more common than previously assumed with prevalence at 4% (Simner et al., 2006). According to Day (2004) the most common form is grapheme-color synaesthesia with prevalence at 68.8% among synaesthetes. Orgasms as inducers for colored visions are considered less frequent with a prevalence of 2.15%. In some cases flavors as synaesthetic concurrents induced by orgasms are mentioned as well. Sexual types of synaesthesia as described below do not only contain orgasms and sexual arousal but also more general tactile inducers such as touching, caressing, and petting (i.e., all forms of physical contact during sexual intercourse). Therefore, sexual synaesthesia actually contains several synaesthetic types. Touches as synaesthetic inducers are mentioned by Day as well. There are described many different concurrents such as colors (4.08%), flavors (1.07%), smell (0.43%), sounds (0.32%) or temperatures (0.11%). Generally, in literature sexual types of synaesthesia are rarely mentioned. Cytowic (1989) notes that in some cases “kissing and sexual intercourse is a reliable trigger (…), causing colored photisms, tactile shapes and textures and tastes”(p. 34). Altogether, this special form of synaesthesia has been neglected so far in scientific research, probably due to the low rate of prevalence and the difficulties in investigating this highly subjective phenomenon.

Outside synaesthesia research, synaesthetic experiences during sexual activity have also been described by sex researchers. Fisher (1970) conducted an interview study to investigate female orgasmic experiences. The words of one subject bear remarkable resemblance to the descriptions by synaesthetes: “(…) the only images I ever experienced at this time and during orgasm is a fuzzy blackness with red or white muted bursts coming through it (…)”(p. 207). The phenomenological description is similar to descriptions of audio-visual forms of synaesthesia as the visualized structures and colors are not static but dynamic and alternating (Haverkamp, 2009). Mosher (1980) refers to this citation as an example of highly extended sexual trance which is defined as “(…) an altered state of consciousness (…) representing a qualitative shift in the pattern of psychological functioning that differs from the norm of alert, waking consciousness” (p. 11). In his model “Three dimensions of depth of involvement in human sexual response” sexual trance is one of three postulated dimensions essential to a maximum extent of depth of involvement which in turn is a condition for efficient sexual stimulation. The maximum extent of sexual trance leads to a state of total absorption. The other dimensions are “role enactment” and “engagement with the partner.” According to Mosher, an equilibrium between those three dimensions is a condition for a maximum extent of depth involvement and thus, for sexual satisfaction. According to Swartz (1994) absorbed states of consciousness—sometimes called altered states of consciousness (ASCs) or sexual trance—are a common phenomenon during sexual arousal and play an important role in sexual responses. Regarding general, inducer-independent criteria for ASCs, Ludwig (1966) postulates synaesthesia may be an important feature of ASCs. Shanon (2003) also discusses the occurrence of synaesthesia in ASCs which seems to be a common phenomenon. Tellegen and Atkinson (1974) who investigates the “high absorption person” describes the inherent ability “to operate diverse representational modalities synergistically so that a full but unified experience is realized” (p. 275) which he claims is a cognitive accomplishment of the absorption trait. He calls this integrative phenomenon “syngnosia” (p. 275), a term used analogous to “synesthesia” which he suggests is one of its components.

Assuming that synaesthesia may be an important constituent of ASCs and that several parallels between synaesthesia and the ability to achieve sexual trance can be found the consequent hypothesis is that synaesthetes with sexual forms of synaesthesia (i.e., synaesthetic perceptions through any sexually experienced inducers) may experience a higher degree of sexual trance during intercourse. The present study investigated this hypothesis by comparing the sexual experiences of a group of synaesthetes with a control group. A further hypothesis concerns the sexual satisfaction of synaesthetes with sexual forms: According to Mosher's (1980) theory that sexual trance is one basic criterion for high sexual involvement, it is assumed that synaesthetes are able to experience a greater sexual satisfaction than a non-synaesthetic standardization sample.

## Materials and methods

### Study participants

Nineteen synaesthetes (17 female, 2 male) were recruited via internet and through an already existing data pool of the synaesthesia work group at the Hannover Medical School (MHH). All subjects were assumed to be psychiatrically and physically healthy which was based on enquiry during acquisition phase. Only synaesthetes who reported synaesthetic perceptions during sexual experiences were included. Synaesthesiae included visual perceptions such as colored shapes. There didn't seem to be any essential differences between the synaesthetic experiences of the male and female subjects i.e., both types of gender described generally similar perceptions. Coexisting synaesthetic forms ranged from emotionally induced ones to more common forms such as grapheme-color synaesthesia which was confirmed via the consistency test. This test was developed to investigate the consistency of mapping across time as it has previously been shown that synaesthetes show greater matching consistency than non-synaesthetes (e.g., when asked to match colors to graphemes and then being re-tested after a delay). It was designed via MatLab (Matrix LABoratory, Version 7.1.) by the MHH synaesthesia research group and is based upon the “synesthesia battery” by Eagleman et al. (2007). The inclusion criterion of a sexual form of synaesthesia was simply confirmed by interviewing the participants, as this form of synaesthesia cannot be verified through any standardized tests. This lack of assurance will be discussed in detail below.

The control group data for the KFSP (Reynolds et al., 1988; Mazer et al., 2000) stem from a data pool of sexual healthy women generated by the Clinical Psychology Department of the MHH. Due to the insufficient number of men in the synaesthetic group, the male participants were excluded from the KFSP research. The mean age of the female synaesthetes (37.06; *SD* ± 11.31) again was not statistically different from that of the controls (36.12; *SD* ± 10.17).

The data of the control group for the OAVAV (Dittrich, 1975, 1998; Passie, 2007) investigation was obtained in a parallel MHH study investigating gender-specific differences in sexual experience. The control group was matched pairwise by gender and age. The mean age of the synaesthetic group (36.53; *SD* ± 11.7) did not differ from the control group (34.2 ± 10.0) as measured by *t*-test.

The study was approved by the MHH Ethics Committee. The subjects were assured that any private information they gave would be kept confidential. By carrying out the procedure via mail the participation was kept anonymous. Each questionnaire was labeled with a code number so that the response rate could be followed. Each participant gave written consent about the participation.

### Psychometric assessment

Two questionnaires were employed. First, the KFSP (“Kurzfragebogen zur Erfassung sexueller Probleme”) is an instrument for the investigation of most relevant aspects of sexual function and dysfunction. The KFSP consists of two different versions; one especially developed for women (KFSP-F) and one for men (KFSP-M) but only the KFSP-F was implemented. The KFSP-F is theoretically based on, but is not a direct translation of the BISF-W (“Brief Index of Sexual Functioning for Women”) which in turn is based on the BSFQ (“Brief Sexual Function Questionnaire for Men”) (Reynolds et al., 1988). The KFSP-F contains four subscales: “Sexual satisfaction,” “sexual appetence,” “arousal/lubrication,” and “orgasm ability.”

Furthermore, the OAVAV was applied to investigate general forms of ASCs. The original version APZ (“Abnorme Psychische Zustände”) is a standardized psychometric assessment of ASCs in humans developed by Dittrich (1975) and is available in English and German (Dittrich, 1998). The APZ consists of three dimensions (“oceanic Boundlessness,” “dread of ego dissolution,” and “visionary restructuralization”). During the process of validation the APZ was revised and renamed the OAV; later it was completed by two more subscales. Thus, the name of the latest version, OAVAV, is a composition of the capital letters representing the five subscales in German: Oceanic boundlessness, dread of ego dissolution, visionary restructuralization, reduction of vigilance, and auditive alterations (Passie, 2007). Each one of the subscales contains specific characteristics of ASCs according to Ludwig (1966). The items have to be rated on a visual analog scale from 0 up to 10. Subjects were instructed to fill out the questionnaire about 10 min after sexual intercourse. The German version of the OAVAV is validated in comprehensive studies (Dittrich, 1998). Independent two-sample *t*-tests were conducted for statistic comparison of the synaesthetic group and the control groups; the statistic analysis was accomplished via PASW (Predictive Analytics Software, version 18). A *p*-value of *p* < 0.05 (α-level) was considered as statistically significant.

Additionally, qualitative semi-structured interviews were conducted with approximately half of the synaesthetic subjects (*n* = 7) who were willing to participate in this part of the study. The interviews were supported by guidelines which were adjusted to the sexual response cycle by Masters and Johnson (1966), in order to investigate perception during different phases of sexual experience. The transcriptions of the interviews were analyzed by the “Grounded Theory” by Glaser and Strauss (1998) which is a systematic methodology to analyze written data. Instead of beginning with a theory, the basic idea is to develop a superordinate theory by extracting key points from the text and then subsequently create codes, concepts, and categories. The results will be presented exemplarily to complement the quantitative results.

## Results

### KFSP-F: sexual function and dysfunction

The KFSP total score was significantly higher in synaesthetes than in the controls, which reflects better sexual function in general. Accordingly, the subscale score for “sexual appetence” was significantly higher (*p* = 0.003) for synaesthetes. Surprisingly, the subscale “sexual satisfaction” was significantly lower for synaesthetes than for control subjects (*p* = 0.036). The subscales “arousal/lubrication” and “orgasm ability” did not reveal any differences between synaesthetes and control group (see Table 1 for details).

**Table 1:** **Sexual function in synaesthetic and non-synaesthetic females**.

**Group comparison by *t*-test for independent samples**							
	**Mean synaesthetic group**	***SD* synaesthetic group**	**Mean control group**	***SD* control group**	***T*-value**	***p***	**Cohen's d**
KFSP-F total	3.27	0.36	2.93	0.36	−2.66	0.01^*^	−0.91
“Sexual appetence” e.g., “How important is it for you in general to have a satisfying sex life?”	3.67	0.77	2.88	0.67	−3.21	0.003^**^	−1.1
“Arousal/ lubrication” e.g., “Do you experience an increasing feeling of arousal during sexual activity?”	2.29	0.75	2.32	0.79	0.11	0.91	0.04
“Orgasm ability” e.g., “How often do you experience an orgasm during sexual intercourse?”	2.37	0.37	2.37	0.32	−1.15	0.26	−0.39
“sexual satisfaction” e.g., “Altogether, how satisfied have you been with your sex life over the last month?”	3.29	1.77	4.66	1.88	2.19	0.04^*^	0.75

Results of the short questionnaire for sexual problems for women (KFSP-F): Comparison between two independent samples by t-test in the appending subscales; significance:

* p < 0.05;

** p < 0.01; degrees of freedom (df) = *32* for all t-tests; effect size according to Cohen's d: Around 0.2 → small effect, around 0.5 → medium effect, around 0.8 and larger values → large effect.

### The OAVAV: altered states of consciousness

Synaesthetes achieved significantly higher scores for total OAVAV, as well as for the subscales “oceanic boundlessness” and “visionary restructuralization” (see Table 2 for details). Since the latter contains visual perceptions as patterns or colors, the high “visionary restructuralization” score for synaesthetes seems to directly reflect their visual synaesthetic perceptions during sexual activity.

**Table 2:** **Altered states of consciousness during sexual intercourse**.

**Group comparison by *t*-test for independent samples**							
	**Mean synaesthetic group**	***SD* synaesthetic group**	**Mean control group**	***SD* control group**	***T*-value**	***p***	**Cohen's d**
OAVAV total	2.3	1.39	1.4	0.85	2.41	0.022^*^	0.78
“Oceanic boundlessness”	3.98	2.09	2.63	1.57	2.24	0.031^*^	0.73
“Visionary restructuralization”	2.83	1.91	1.08	1.03	3.49	0.001^**^	1.32
“Dread of ego dissolution”	0.56	0.51	0.77	0.83	−0.96	0.346	−0.31
“Reduction of vigilance”	2.5	1.84	1.58	1.56	1.67	0.104	0.54
“Auditive alterations”	0.91	1.47	0.3	0.29	1.78	0.084	0.57

Results of the altered states of consciousness questionnaire (OAVAV): Comparison between two independent samples by t-test in the appending subscales; significance:

* p < 0.05;

** p < 0.01; degrees of freedom (df) = *34* for all t-tests; effect size according to Cohen's d: Around 0.2 → small effect, around 0.5 → medium effect, around 0.8 and larger values → large effect.

### Personal interviews: the sexual response cycle in synaesthetes and personal significance of synaesthetic perceptions

A detailed qualitative analysis according to the “Grounded Theory” by Glaser and Strauss (1998) could only be accomplished for three of the interviewed subjects, because the other participants would not comply with being recorded on tape during the interview, due to the intimate subject. By analyzing the resulting transcriptions two main categories could be extracted: (1) stages of sexual experience and (2) significance of the synaesthetic perceptions.

Concerning the first category, the synaesthetes reported different stages of synaesthetic experiences analogous to the different stages of the sexual response cycle by Masters and Johnson (1966). Not all synaesthetes were capable of allocating their synaesthetic perceptions to one of the different stages but variation in the appearance of their perceptions over time was observed in every subject. Table 3 shows the five different stages of the response cycle [including the desire stage added by Kaplan (1974)] with exemplary citations.

**Table 3:** **Exemplary citations by the synaesthetes questioned about the different phases of the human sexual response cycle**.

**Phase**	**Characteristics**	**Exemplary citations**
0. Appetence phase	Sexual fantasies, development of sexual drive	“This phase has an orange character”
1. Excitement phase	Increment of sexual drive, initiation of multiple physical reactions (e.g., increase of breathing rate, rise of blood pressure, sex flush)	“(…) and it's getting more intensive (…) starting with few colors at the beginning it becomes more and more intense (…)”
2. Plateau phase	Enhancement of reactions during the excitement phase	“The greater the excitement becomes the more the thoughts are canalized” “The initial fog transforms into a wall”
3. Orgasmic phase	Conclusion of plateau phase; resolution of vasocongestion and myotonia	“Then it's like that: In the moment of orgasm the wall bursts (…) ringlike structures (…) in bluish—violet tones”
4. Resolution phase	Resolution of arousal as retrograde progression	“The resolution phase varies between pink and yellow”

Concerning the second category, a significant emotional compound of sexual synaesthesia was detected: All of the synaesthetes questioned described themselves as “emotional synaesthetes” [which is based upon the term “Gefühlssynästhesie” by Emrich (2009)], generally experiencing synaesthetic perceptions induced by emotional states. The accompanying emotions during each state of sexual excitement seemed to influence the synaesthetic perceptions. The degree of emotional influence on the synaesthetic perceptions can be illustrated by the example of one female synaesthete who describes her desire stage as orange while claiming that she experiences orange itself as quite aversive. On further enquiry, the subject admitted that her aversive perception during the desire stage is based upon a more general aversion to sexuality which she describes as low interest in sexual activity. Sexuality never played an important role in her life and this previously used to concern her when she compared herself with others. It is noteworthy that the synaesthetes reported almost consistently that they consider the synaesthetic experiences as enriching for themselves, but that at the same time it makes them feel isolated from their environment during sexual intercourse.

## Discussion

By studying synaesthesia we can learn a lot about consciousness in general (e.g., different types of synaesthesia can provide us with clues for theories concerning the nature of consciousness). ASCs—such as synaesthesia—are a part of human consciousness and as such, they need to be studied in order to understand its nature (Sagiv and Frith, 2013).

Based on the various indications that synaesthesia and sexual trance seem to share certain phenomenological characteristics, we hypothesized an increased degree of sexual trance and sexual satisfaction in synaesthetes. Indeed, synaesthetes depicted significantly higher levels of “oceanic boundlessness” and “visionary restructuralization” reflecting sexual trance and visual synaesthesia during sexual intercourse. However, contrary to our hypotheses, synaesthetes were less satisfied with their sexuality, possibly due to a lack of partner involvement regarding synaesthetic perceptions.

Regarding the possible neurobiological mechanisms of synaesthetic perceptions during sexual intercourse, an imbalance between prefrontal and limbic brain function may be an important factor. Some ASCs can be accompanied by transient frontal hypoactivity as made evident by Dietrich (2003). This overlaps with a former theory by Cytowic (1989), who proposed a limbic basis for synaesthesia due to insufficient inhibition of subcortical activity by cortical processes, which was supported by tentative neurophysiological data from Schiltz et al. (1999). In a more recent study by Cohen-Kadosh et al. (2009) it was possible to induce synaesthetic experiences through hypnotic states which are also accompanied by transient changes in prefrontal functioning. Passie et al. (2004) suggested that hyperventilation during sexual intercourse may be a mechanism that intensifies the sexual experience due to cortical, in particular frontal inhibition, which can be seen as congruent with the assumption that synaesthetes are able to experience a deeper state of trance. This possible neurobiological link between synaesthetic proneness and intensification of sexual trance may be an explanation for the higher values for synaesthetes in the ASCs questionnaire although the present study certainly remains hypothetical on this topic due to the lack of actual neurophysiological data.

Concerning the postulated increased sexual satisfaction of synaesthetes, the employment of the KFSP-F showed a significantly lower level of sexual satisfaction in synaesthetes and significantly higher values in the subscale “sexual appetence” and the total KFSP-F score. Apparently the female synaesthetes were less satisfied despite increased sexual appetence and a possibly heightened sexual trance. One possible explanation for these, at first sight, contradictory results can be found in the theory by Mosher (1980) who postulates sexual trance as one of the three dimensions essential to maximum extent of depth of sexual involvement. The model proposes that equilibrium between the three dimensions is mandatory for sexual satisfaction, whereas over-emphasis of one dimension develops an assimilating effect and averts satisfaction. In the case of over-emphasis of the dimension “sexual trance” the other dimensions—“role enactment” and “engagement with the partner”—are neglected. The person becomes more introverted and separated from the environment which may impair partner involvement. Thus, the high degree of sexual trance in synaesthetes may provoke an increased focus on sensual experiences on the inside, instead of partner-related aspects. This explanation is supported by the results of the personal interviews in which the synaesthetes reported an isolating effect of their synaesthetic experiences, while at the same time they experienced their perceptions as enriching and pleasant for themselves.

We would like to emphasize that the results are restricted by some methodological limitations. First of all—as has already been noted above—the presence of sexual synaesthesia could not be confirmed via consistency test [a test to investigate the consistency of mapping across time according to the “synaesthesia battery” by Eagleman et al. (2007)]. Therefore, the results presented should be treated with care.

This lack of evidence is due to the fact that sexual synaesthesia obviously cannot be tested in laboratory conditions by using the original inducers. However, it is still outstanding if there is a possibility of testing this form of synaesthesia without the original inducer which would imply a more semantic nature of the stimuli (i.e., ideaesthesia) (Jürgens and Nikolic, 2012). Concerning the question whether or not sexual synaesthesia could be considered as ideaesthesia, the reports of the interviewed synaesthetes show contradictory results. Thus, some of them reported more sensory based inducers which seem directly connected to perceptual stimuli (e.g., caressing). For them it was consequently difficult during the interview to remember the exact appearance of their concurrents by memory. Others, on the contrary, reported a more conceptual way in experiencing their synaesthetic concurrents, for example the female synaesthete who experiences her sexual desire stage in orange and who was easily able to visualize her synaesthetic experiences by memory. In summary, it still remains unclear whether or not sexual synaesthesia can be considered as ideaesthesia. Certainly, in case it could be confirmed, it would give an opportunity to prove in a laboratory setting that this form of synaesthesia really exists. This question should be topic of further enquiry.

However, it should be noted that, in our sample, other varieties of synaesthesia, such as grapheme-color or audio-visual forms, could be confirmed via consistency test which can be considered as clue for the credibility of the subjects. Another clue is that the “visionary restructuralization” scale showed significantly higher values for the synaesthetes than for controls, reflecting the general existence of visual synaesthesiae in our subjects.

Finally, it has to be pointed out that this study can be considered as a pilot project providing clues for further investigation. The descriptive character of some of the methods seems to be fairly adequate for this primary examination. Further research on this issue is still outstanding.

## Conclusion

The present investigation reveals novel aspects of ASCs in synaesthetes; synaesthetic experiences during sexual arousal seem to be accompanied by a higher degree of sexual trance consisting of an experienced loss of environmental boundaries and varying visual perceptions. This increment in trance possibly leads to accelerated introversion and a shift in attentional resources focusing exclusively on inner perceptions. Whether or not this heightened introversion attenuates the partner involvement—and consequently the synaesthete's own sexual satisfaction—should be the subject of further research.

### Conflict of interest statement

The authors declare that the research was conducted in the absence of any commercial or financial relationships that could be construed as a potential conflict of interest.

## References

[B1] Cohen-Kadosh R. Henik A. Catena A. Walsh V. Fuentes L. J. (2009). Induced cross-modal synaesthetic experience without abnormal neuronal connections. Psychol. Sci. 20, 258–265 10.1111/j.1467-9280.2009.02286.x 19175754

[B2] Cytowic R. E. (1989). Synesthesia: A Union of the Senses. New York, NY: Springer

[B3] Day S. (2004). Some demographical and docio-cultural aspects of synesthesia, in Synesthesia: Perspectives from Cognitive Neuroscience, ed Sagiv D. (New York, NY: Oxford University Press), 11–33

[B4] Dietrich A. (2003). Functional neuroanatomy of altered states of consciousness: the transient hypofrontality hypothesis. Conscious. Cogn. 12, 231–256 10.1016/S1053-8100(02)00046-6 12763007

[B5] Dittrich A. (1975). Zusammenstellung eines Fragebogens (APZ) zur Erfassung abnormer psychischer Zustände. Z. Klin. Psychol. Psych. 23, 12–20

[B6] Dittrich A. (1998). The standardized psychometric assessment of altered states of consciousness (ASCs) in humans. Pharmacopsychiatry 31 Suppl. 2, 80–84 10.1055/s-2007-979351 9754838

[B7] Eagleman D. M. Kagan A. D. Nelson S. S. Sagaram D. Sarma A. K. (2007). A standardized test battery for the study of synesthesia. J. Neurosci. Methods 159, 139–145 10.1016/j.jneumeth.2006.07.012 16919755 PMC4118597

[B8] Emrich H. M. (2009). Was bedeutet Gefühlssynästhesie? in Synästhesie der Gefühle Tagungsband zur Konferenz “Die fröhlichen Sieben” Synästhesie, Personifikation und Identifikation, ed Sinha J. (Hannover: Synaisthesis Verlag), 15–18

[B9] Fisher S. (1970). The Female Orgasm: Psychology, Physiology, Fantasy. New York, NY: Basic Books

[B10] Glaser B. G. Strauss A. L. (1998). The Discovery of Grounded Theory: Strategies of Qualitative Research. Chicago, IL: Aldine

[B11] Haverkamp M. (2009). Music and motion - Alexander Truslit and the research on synesthesia, in Synästhesie der Gefühle Tagungsband zur Konferenz “Die fröhlichen Sieben”: Synästhesie, Personifikation und Identifikation, ed Sinha J. (Hannover: Synaisthesis Verlag), 135–159

[B12] Jürgens U. M. Nikolic D. (2012). Ideaesthesia: conceptual processes assign similar colours to similar shapes. Transl. Neurosci. 3, 22–27 10.2478/s13380-012-0010-4

[B13] Kaplan H. S. (1974). The New Sex Therapy. New York, NY: Brunner/Mazel

[B14] Ludwig A. M. (1966). Altered states of consciousness. Arch. Gen. Psychiatry 15, 225–234 10.1001/archpsyc.1966.01730150001001 5330058

[B15] Masters W. H. Johnson V. E. (1966). Human Sexual Response. Toronto, ON; New York, NY: Bantam Books

[B16] Mazer N. A. Leiblum S. R. Rosen R. C. (2000). The brief index of sexual functioning for women (BISF-W): a new scoring algorithm and comparison of normative and surgically menopausal populations. Menopause 7, 350–363 10.1097/00042192-200007050-00009 10993034

[B17] Mosher D. L. (1980). 3 Dimensions of depth of involvement in human sexual-response. J. Sex Res. 16, 1–42 10.1080/00224498009551060

[B18] Passie T. (2007). Bewusstseinszustände: Konzeptualisierung und Messung. Münster: LIT Verlag

[B19] Passie T. Wagner T. Hartmann U. Schneider U. Emrich H. M. (2004). Acute hyperventilation syndromes induced by sexual intercourse: evidence of a psychophysical mechanism to intensify sexual experience? Arch. Sex. Behav. 33, 525–526 10.1023/B:ASEB.0000044736.73344.c2 15483366

[B20] Reynolds C. F. 3rd. Frank E. Thase M. E. Houck P. R. Jennings J. R. Howell J. R. (1988). Assessment of sexual function in depressed, impotent, and healthy men: factor analysis of a brief sexual function questionnaire for men. Psychiatry Res. 24, 231–250 10.1016/0165-1781(88)90106-0 3406241

[B21] Sagiv N. Frith C. D. (2013). Synesthesia and consciousness, in Oxford Handbook of Synesthesia, eds Simner J. Hubbard E. M. (Oxford: Oxford University Press), 924–940

[B22] Schiltz K. Trocha K. Wieringa B. M. Emrich H. M. Johannes S. Munte T. F. (1999). Neurophysiological aspects of synesthetic experience. J. Neuropsychiatry Clin. Neurosci. 11, 58–65 9990557 10.1176/jnp.11.1.58

[B23] Shanon B. (2003). Three stories concerning synaesthesia a commentary on Ramachandran and Hubbard. J. Conscious. Stud. 10, 69–74

[B24] Simner J. Mulvenna C. Sagiv N. Tsakanikos E. Witherby S. A. Fraser C. (2006). Synaesthesia: the prevalence of atypical cross-modal experiences. Perception 35, 1024–1033 10.1068/p5469 17076063

[B25] Swartz L. H. (1994). Absorbed states play different roles in female and male sexual response: hypotheses for testing. J. Sex Marital Ther. 20, 244–251 10.1080/00926239408403434 7996595

[B26] Tellegen A. Atkinson G. (1974). Openness to absorbing and self-altering experiences (“absorption”), a trait related to hypnotic susceptibility. J. Abnorm. Psychol. 83, 268–277 10.1037/h0036681 4844914

